# Comparative Effects of Crocin and Losartan on RAGE, TGF‐β, TNF‐α Gene Expression and Histopathological Changes of the Liver Tissue in Rats With Diabetes

**DOI:** 10.1002/edm2.70016

**Published:** 2024-11-28

**Authors:** Shahnaz Rajabi, Yaser Mohammadi, Hamid Kabiri‐rad, Mahdiyeh Rajabi‐moghaddam, Azam Rezaei Farimani

**Affiliations:** ^1^ Student Research Committee Birjand University of Medical Sciences Birjand Iran; ^2^ Department of Clinical Biochemistry, School of Medicine Birjand University of Medical Sciences Birjand Iran; ^3^ Department of Biochemistry, School of Medicine Iran University of Medical Sciences Tehran Iran; ^4^ Cellular and Molecular Research Center Birjand University of Medical Sciences Birjand Iran; ^5^ Department of Pathology, School of Medicine Birjand University of Medical Sciences Birjand Iran

**Keywords:** crocin, diabetes, inflammatory markers, liver disease, losartan, Oxidative stress

## Abstract

**Background and Objectives:**

AGEs, via RAGE, increase the development of hyperglycemia‐induced liver damage, and blocking this axis is associated with a reduction in liver disease progression. The goal of this study was to determine how crocin and losartan influenced RAGE, TNF‐α and TGF‐β gene expression in diabetic rats, as well as histological changes in liver tissue.

**Materials and Methods:**

Diabetes was induced in 40 male Wistar rats using Streptozotocin (50 mg/kg, IP). There were five groups of rats: diabetic and healthy groups, diabetic rats given crocin (50 mg/kg), losartan (25 mg/kg) and both (crocin + Los). Serum glucose, ALT and AST levels were measured 4 weeks later. qPCR was used to examine the TNF‐α, TGF‐β and RAGE gene expression in liver tissue.

**Results:**

Crocin was found to be effective in lowering FBG in the diabetes group. The serum levels of ALT and AST decreased in all treated groups, but this decrease was significant in the crocin + Los group (*p* < 0.05). The relative expression of RAGE, TNF‐α and TGF‐β genes was significantly higher in the diabetes group compared to the healthy group. The expression of these genes decreased in groups treated with crocin and Losartan compared to the diabetes group. The highest reduction in RAGE and TGF‐β gene expression was reported in those treated with crocin + Los. Histopathology results showed that the diabetes group had more bile ducts and necrosis than the healthy control group, which had no tissue changes. Hepatocyte degeneration, bile duct proliferation, inflammatory changes and hepatocyte necrosis were mild in the treated groups, but no hepatocyte necrosis was observed in the crocin + Los group.

**Conclusion:**

Crocin may be a feasible therapeutic agent for treating diabetes and its symptoms when combined with pharmaceutical medications. Human research is still needed to reach clear conclusions.

## Graphical abstract

Crocin and losartan can improve liver function by reducing oxidative stress and inflammation.

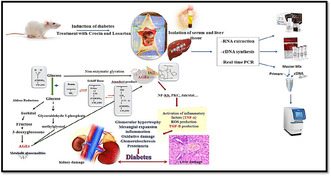

## 1 Introduction

Diabetes mellitus (DM), a metabolic disorder, is recognised as a critical public health concern with serious consequences for human life and healthcare expenses. It is responsible for 1.5 million annual fatalities around the world [1]. People with DM are at greater risk of liver problems including non‐alcoholic fatty liver disease, liver enzyme deficiency, cirrhosis, acute liver failure (ALF) and hepatocellular carcinoma [2]. Hyperglycemia accelerates the formation of altered proteins (Amadori's product) and advanced glycation end products (AGEs) [3]. The RAGE (receptor for AGE) is the main mechanism by which the AGEs influence a range of cell processes, including inflammation, proliferation, apoptosis, angiogenesis, migration and fibrosis. RAGE is expressed by hepatocytes, stellate cells and hepatoma cells. Numerous studies show that activating RAGE with various ligands causes oxidative stress, which in turn activates the downstream RAGE pathway in the liver, causing the occurrence and progression of numerous liver disorders. Based on prior studies, TNF‐α (Tumour necrosis factor alpha) and TGF‐β (Transforming growth factor beta) are indicative of inflammation and fibrosis in patients with liver disease. These cytokines can upset the equilibrium between extracellular matrix breakdown and synthesis and cause apoptosis by altering ECM proteins and NF‐κb (Nuclear factor kappa B) and activating caspases [4, 5, 6, 7, 8, 9]. These cytokines are upregulated by the AGE–RAGE axis. Therefore, diabetes‐related liver disorders can be prevented or treated by blocking the RAGE signalling pathway [10, 11]. Losartan (Los) is a chemical drug with antioxidant defence properties that is used to treat diabetes [12]. Los, an Angiotensin II receptor antagonist, reduces the activity of TGF‐β and delays the progression of liver fibrosis [13]. Due to the negative side effects of chemical medications, research has focused on herbal remedies. Crocin, a carotenoid responsible for the colour of saffron, has a wide range of therapeutic and medicinal benefits, including anti‐inflammatory, anti‐glycaemic and antioxidant. There is proof that crocin protects against metabolic syndrome, obesity and DM [14, 15]. According to studies on the effective role of crocin in diabetes and reducing its complications, the goal of this study is to investigate the comparative effect of crocin and Los on RAGE, TGF‐β and TNF‐α gene expression in liver tissue.

## 2 Materials and Procedures

### 2.1 Chemicals and Drugs

Streptozotocin (STZ) (Product No. S0130) and Crocin (Product No.17304) were obtained from Sigma‐Aldrich (St Louis, MO, USA). Losartan (Product No.146‐L3) was provided by ACTOVERCO Pharmaceutical Company (Karaj, Iran). Crocin and Losartan were dissolved in distilled water and administered orally.

### 2.2 Animals

40 Wistar male rats were purchased from Birjand University of Medical Sciences Experimental Research Centre and housed under standard conditions with free access to sufficient food and water, a temperature of 25°C ± 2°C, and an appropriate habitat. The study was carried out in compliance with the protocols for the care and use of laboratory animals and were authorised by the Birjand University of Medical Sciences Ethics Committee (IR.BUMS.REC.1401.038).

### 2.3 Induction of Diabetes

Type 1 diabetes was induced via a single intraperitoneal injection of 50 mg/kg STZ (solution in 0.1 mM citrate buffer at pH 4.5). To prevent hypoglycaemia after injection, rats had free access to a glucose solution. To validate the induction of diabetes, a glucometer (Accu‐Chek Active) was used to measure fasting blood glucose (FBG) after 72 h. Diabetes was regarded as having an FBG level over 250 mg/dL. Animals were housed separately in groups of eight and randomly assigned to one of five experimental groups. Normal rats served as healthy controls and diabetic rats served as diabetic controls, diabetic rats received crocin, diabetic rats received Los and diabetic rats received crocin + Los.

### 2.4 Treatment

Treatment with crocin (50 mg/kg) and Los (25 mg/kg) was started 28 days after the induction and progression of diabetes. Los and crocin were administered orally by gavage after dissolving in distilled water for 4 weeks. The administrated drug doses used in our study were based on previous research and established protocols [16].

### 2.5 Sampling

The animals were anaesthetised at the end of the treatment period using ketamine and xylazine (50 + 20 mg/kg, respectively). Blood was drawn from the animal's heart to run biochemical testing. The serum was isolated and kept at −20°C. The liver was placed in liquid nitrogen after being washed with cold normal saline and stored at −80°C.

### 2.6 Measuring of FBG, ALT and AST

Serum levels of FBG, ALT (Alanine Transaminase) and AST (Aspartate Transaminase), were determined using a kit (Product No. 9897956, Pars Azmun Company—Iran) by Prestige 24i autoanalyser.

### 2.7 Gene Expression Analysis Using Real‐Time PCR (qPCR)

Frozen liver tissues (0.05 g) were homogenised using liquid nitrogen. Total RNA was extracted using Kiazol reagent (Product No.011001, KIAZIST, Iran) according to the manufacturer's instructions. The isolated RNA was examined using a 1.5% agarose gel. A nanodrop ultraviolet (UV) spectrophotometer (Biotech‐America) was also used to measure the quantity and purity of RNA. Following the evaluations, cDNA synthesis was performed using a kit (Product No. A101158, Pars Tos, Iran). The device (Applied Biosystems, USA) and the 2× SYBR Green Real‐time PCR master mix (Product No. C101017, Pars Tous, Iran) were then used to analyse gene expression. One cycle at 95°C for 10 min, 40 cycles at 95°C for 15 s, 65°C for 30 s and 72°C for 30 s comprised the amplification operation. The Beta‐actin gene was used as an internal control gene. The relative fold gene expression of the samples was evaluated using the delta–delta Ct technique (2^−∆∆Ct^). All qPCR reactions were performed in duplicate. Table 1 contains primer information.

**TABLE 1:** Primer sequences for Real‐time PCR.

Genes	Primer sequence
Beta‐Actin	Forward: 5'‐CGCGAGTACAACCTTCTTGC‐3' Reverse: 5'‐GTCTACAACATGATCTGGGTCA‐3'
TNF‐α	Forward: 5'‐CAAATGGGCTCCCTCTCATC‐3' Reverse: 5'‐GCTTGGTGGTTTGCTACGAC‐3'
TGF‐β	Forward: 5'‐GCAACAATTCCTGGCGTTAC‐3' Reverse: 5'‐GTATTCCGTCTCCTTGGTTCAG‐3'
RAGE	Forward: 5'‐CTCACCCATGCAAGGATTCA‐3' Reverse: 5'‐AATACAACAAAACCCGCAGC‐3'

### 2.8 Histopathology of Liver Tissue

Liver tissues were carefully excised and immediately fixed in 10% neutral‐buffered formalin to preserve tissue morphology. After a 24‐h fixation period, the samples were processed through a series of ethanol solutions with increasing concentrations (70%, 80%, 90% and 100%) for dehydration, followed by clearing in xylene. The dehydrated tissues were then embedded in paraffin blocks. Sections of 4–5 μm thickness were cut from the paraffin‐embedded liver tissues using a rotary microtome (Leica RM2235). These thin sections were mounted on glass slides, deparaffinised with xylene and rehydrated through descending concentrations of ethanol (100%, 90%, 80%, 70%) to water. To assess general liver architecture and detect pathological changes, the tissue sections were stained using Haematoxylin and Eosin (H&E) staining. The stained slides were examined under a microscope (Olympus‐B × 41, Japan). Liver histopathology was evaluated based on several criteria, including the presence of hepatocellular degeneration, necrosis, inflammation and any evidence of fibrosis or bile duct proliferation. Pathological changes were scored semi‐quantitatively using a grading system ranging from 0 (no damage) to 4 (severe damage) by two independent pathologists who were blinded to the treatment groups [17].

### 2.9 Statistical Evaluation of Data

The Shapiro–Wilk test was used to analyse the normality distribution of the data. A one‐way analysis of variance (ANOVA) with Tukey's post hoc test was used to assess data that were normally distributed. For each group before and after compassion, the paired sample t‐test was performed. The mean and standard deviation were used to present all data. *p* < 0.05 was regarded as statistically significant. The analysis was carried out by IBM SPSS Statistics 16.0 software (SPSS Inc., Chicago, IL).

## 3 Results

### 3.1 Crocin and Los' Impact on Biochemical Parameters

#### 3.1.1 FBG Changes in the Study Groups

The FBG levels before and after the intervention are shown in Table 2. There were no significant changes in FBG levels between the groups before diabetes induction (*p* > 0.05). FBG levels were considerably greater in the diabetes groups compared to the healthy control group on 28 days (day 28) after the development of hyperglycaemia (*p* < 0.001). Intergroup comparisons following the intervention (day 56) demonstrated that crocin treatment significantly reduced FBG levels in contrast to the diabetes group (*p* < 0.001). When compared to the diabetic control group, FBG levels in the Los and crocin + Los groups were lower, but these changes were not statistically significant. Intra‐group comparisons preceding and following the intervention revealed that FBG levels rose in the diabetes control group and decreased in the treated groups, however, they were not significantly different.

**TABLE 2:** Crocin and Los' impact on FBG levels.

Days	Study groups(mg/dL)				
	Healthy	Diabetic	Crocin	Los	Crocin + Los
0th	84.5 ± 9.8	96.25 ± 5.7	88.50 ± 6.9	93.85 ± 8.3	95.714 ± 5.3
28th	89.87 ± 5.9	407.00 ± 48.4*	387.38 ± 17.9*	398.71 ± 79.54*	392.57 ± 59.5*
56th	91.50 ± 2.8	433.14 ± 53.2*	336.63 ± 38.26*, ^#^	386.43 ± 41.1*	374.14 ± 41.4*
** *p*	0.17	< 0.001	< 0.001	< 0.001	< 0.001

*Note:* Data are presented as Mean ± SD. Changes of FBG in groups of healthy, diabetic, crocin (Diabetic treated with crocin), Los (Diabetic treated with losartan), crocin + Los (Diabetic treated with losartan & crocin), *n* = 8.

^#^ *p* < 0.05: significant differences versus diabetic group.

** *p*‐value: intragroup analysis on different study days.

* *p* < 0.05: significant differences versus the healthy control group.

#### 3.1.2 ALT And AST Changes in the Study Groups

The diabetic group's serum levels of ALT and AST increased significantly when compared to the healthy control group, according to the results of ANOVA and Tukey's post‐hoc test (*p* = 0.001). The serum levels of ALT and AST were also lower in the treated groups than in the diabetic control group, and this decrease was statistically significant in the crocin + Los group (*p* = 0.001). Serum levels of ALT and AST also decreased in the Crocin and Los groups, but it was not significant (Table 3).

**TABLE 3:** The effect of crocin and Los on serum ALT and AST levels.

Serum levels	Study groups				
	Healthy	Diabetic	Crocin	Los	Crocin + Los
ALT (U/L)	50.66 ± 6.25	110 ± 12.49*	102.50 ± 27.66	106.28 ± 49.37	79.85 ± 18.56^#^
AST (U/L)	88.50 ± 18.67	142.14 ± 44.15*	121.25 ± 14.43	133.57 ± 42.45	111.14 ± 17.38^#^

*Note:* Data are presented as Mean ± SD. ALT and AST changes in healthy, diabetic, crocin (diabetic treated with crocin), Los (diabetic treated with losartan), crocin + Los (diabetic treated with losartan and crocin), *n* = 8.

* *p* < 0.05: significant difference compared to the healthy control group.

^#^ *p* < 0.05: significant difference compared to the diabetic group.

### 3.2 Los and Crocin's Effects on Gene Expression

#### 3.2.1 TGF‐β Relative Expression Changes

**FIGURE 1:**
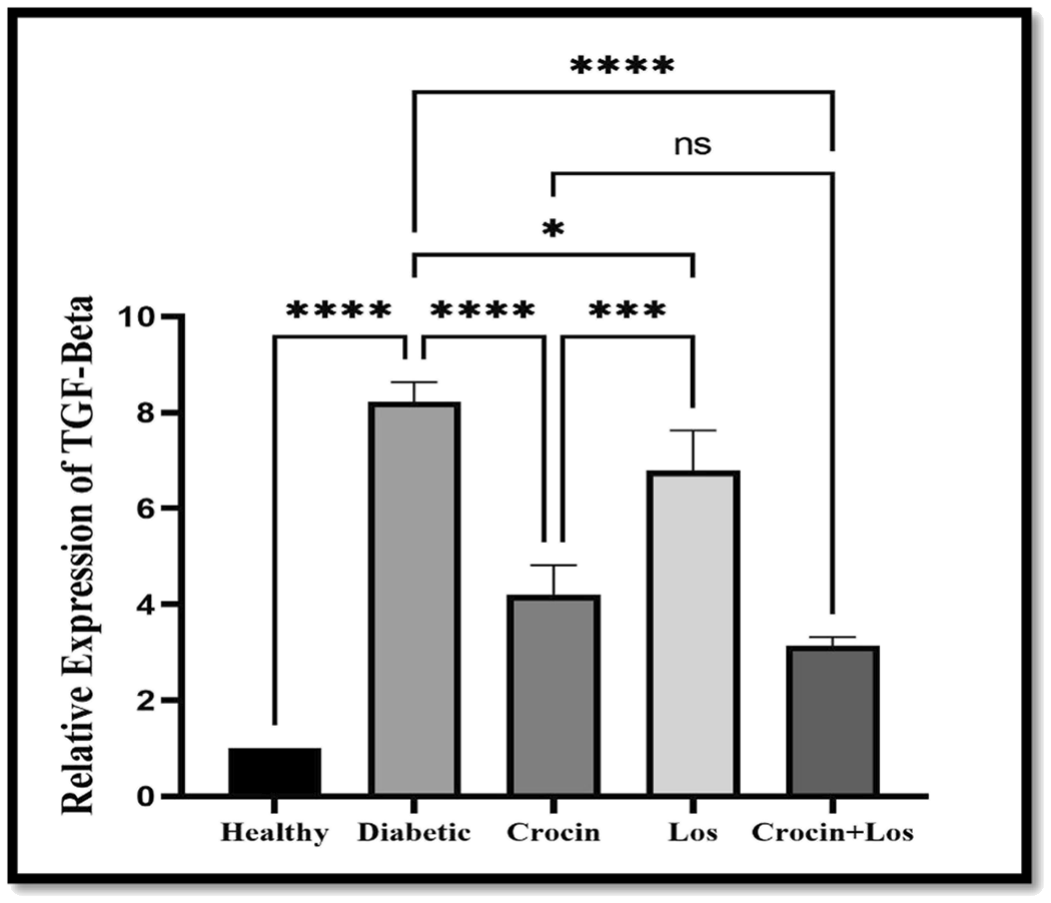
The effect of crocin and Los on TGF‐β gene expression in the liver. Data are presented as Mean ± SD. TGF‐β changes in healthy, diabetic, crocin (diabetic treated with crocin), Los (diabetic treated with losartan), crocin + Los (diabetic treated with losartan and crocin), *n* = 8. *p = 0.03, ****p < 0.0001. A significant decrease in TGF‐β gene expression was observed in the groups treated with crocin and Los (***p = 0.0001) vs. diabetes group. Also, a decrease in TGF‐β expression was seen in the group that received crocin + Los, but this decrease was not significant(ns) compared to the two treated groups, but this reduction was significant compared to the diabetes group.

At the mRNA levels, measuring the relative expression of TGF‐β in the liver (Figure 1), revealed a significant rise in the diabetic control group compared to the healthy group (*p* < 0.0001) and a decrease in the crocin + Los and crocin groups (*p* < 0.0001).

#### 3.2.2 RAGE Relative Expression Changes

**FIGURE 2:**
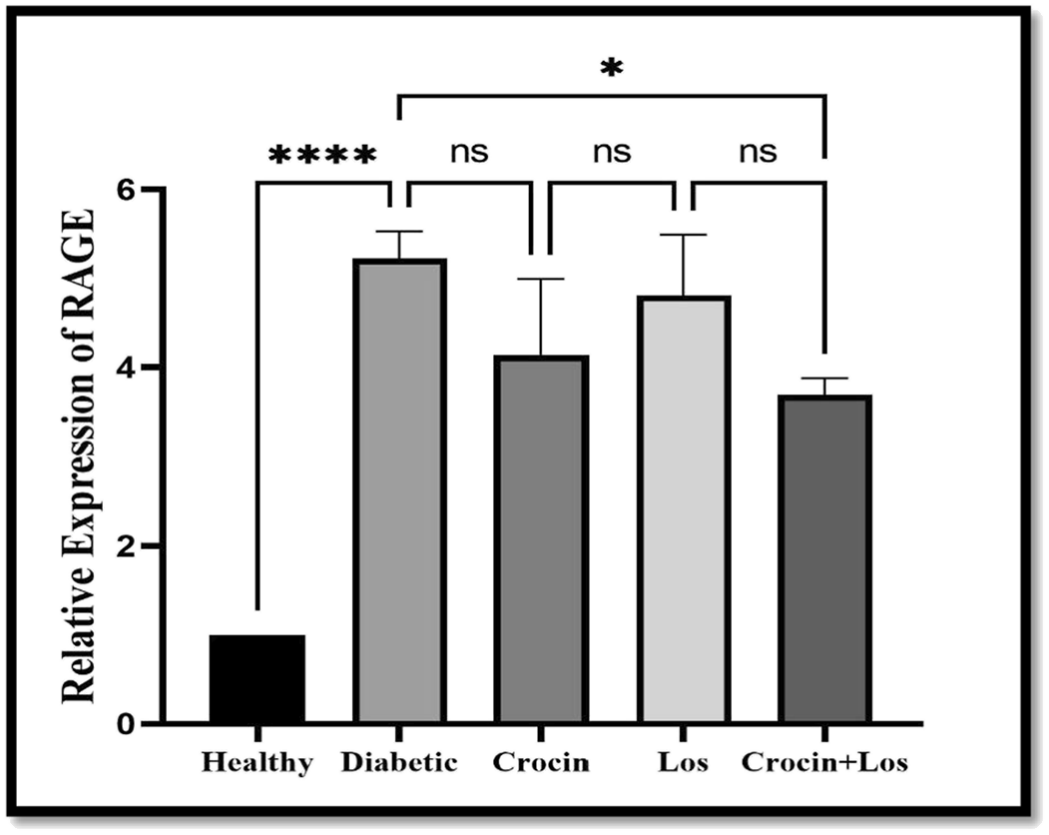
The effect of crocin and Los on RAGE gene expression in the liver. Data are presented as Mean ± SD. RAGE changes in healthy, diabetic, crocin (diabetic treated with crocin), Los (diabetic treated with losartan), crocin + Los (diabetic treated with losartan and crocin), *n* = 8. The figure shows an increase in gene expression in the diabetic group compared to the healthy group (****p < 0.0001). In the treated groups, the increase in the level of RAGE was less, but in the group that received the combination of two drugs, the decrease in RAGE expression was higher (*p = 0.02).

Our findings revealed a significant increase in RAGE gene expression in the diabetes control group as compared to the healthy group (*p* < 0.0001). The expression of this gene was considerably lower in the crocin + Los group compared to the other treatment groups (*p* = 0.02), as shown in Figure 2.

#### 3.2.3 TNF‐α Relative Expression Changes

**FIGURE 3:**
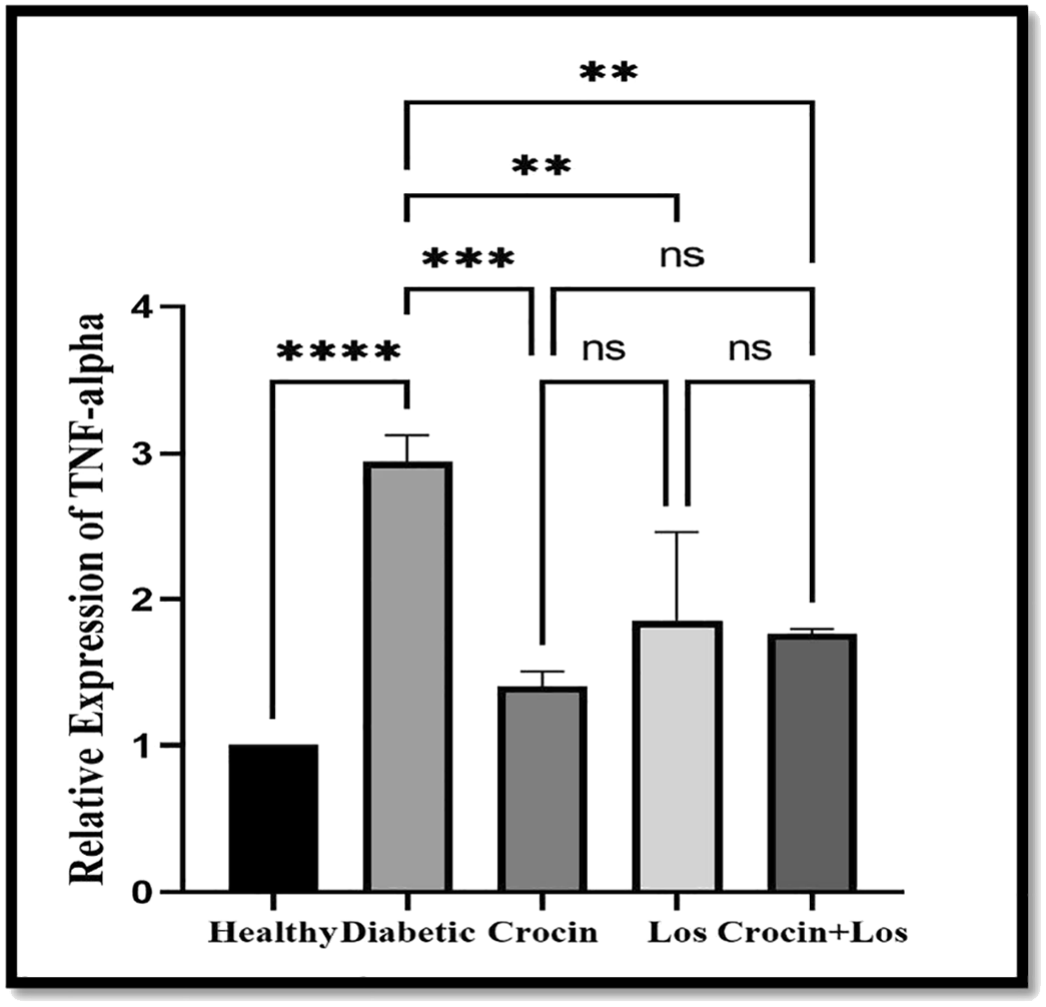
The effect of crocin and Los on TNF‐α gene expression in the liver. Data are presented as Mean ± SD. TNF‐α changes in healthy, diabetic, crocin (diabetic treated with crocin), Los (diabetic treated with losartan), crocin + Los (diabetic treated with losartan and crocin), *n* = 8. Ns means it's not significant. An increase in TNF‐α expression has been shown in the diabetic group compared to the healthy group (****p < 0.0001). The expression of TNF‐α decreased in all treated diabetic groups compared to the untreated diabetic group (**p = 0.006), which was more pronounced in crocin group (***p = 0.0005).

In Figure 3, the relative changes of TNF‐α gene expression in the liver tissue of the studied groups are presented. We observed an increase in gene expression in the diabetic group compared to the healthy group. Gene expression decreased in other treated groups, although the relative expression of TNF‐α in the crocin group was lower than in other groups versus the diabetes group.

### 3.3 The Impact of Crocin and Los on the Liver Tissue's Histopathology

**FIGURE 4:**
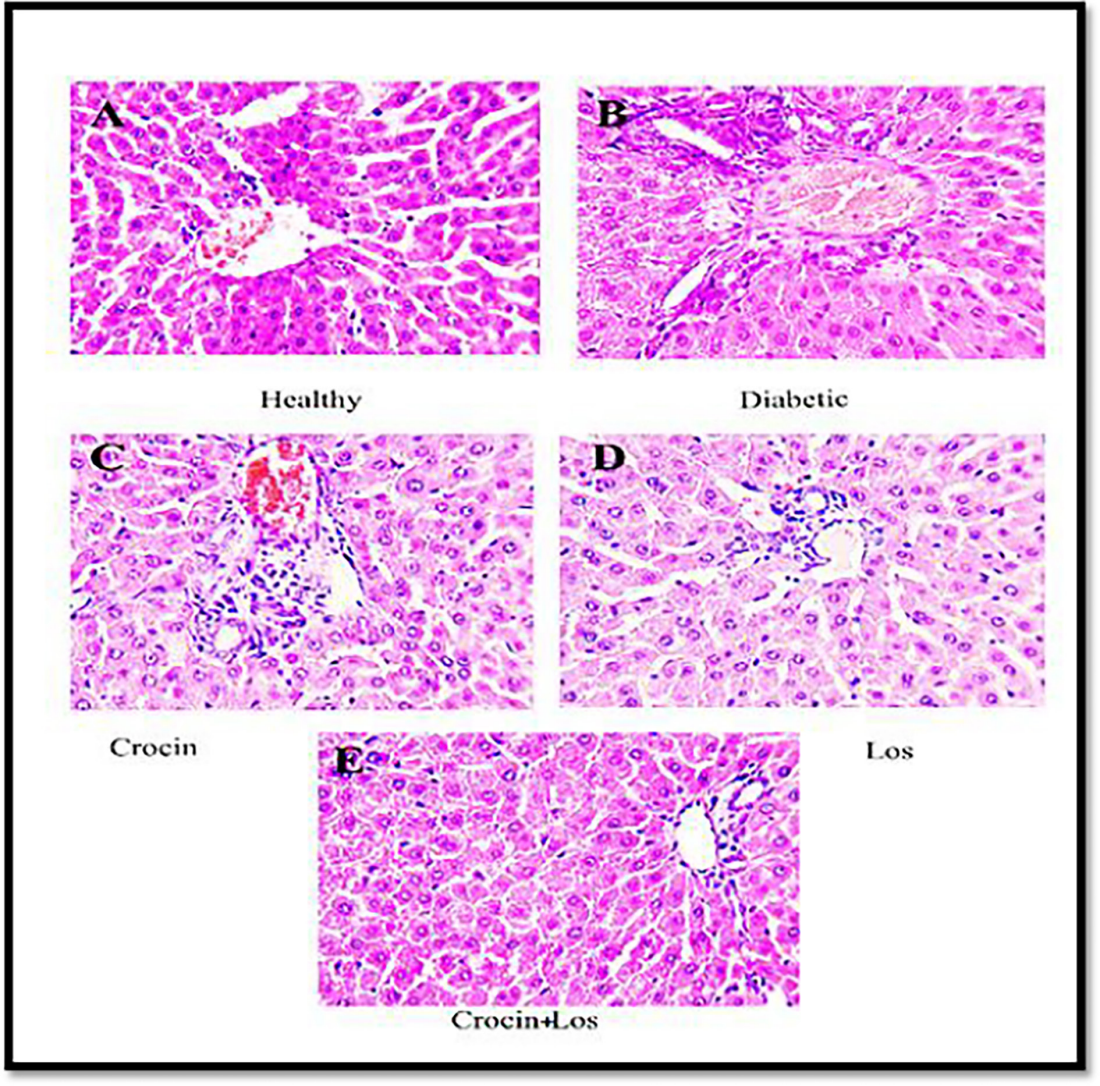
Liver tissue histopathological in the study groups. Hepatic degeneration, proliferation of bile ducts, inflammatory changes and necrosis of liver cells were amongst the most important tissue changes observed in the diabetes group. These changes are less noticeable in other groups. The crocin + Los group did not exhibit hepatocyte necrosis. 400× magnification, H&E colouring.

The severity of alterations in Table 4 was used to rate and score the histological examination. The healthy group's liver had an intact and homogeneous histoarchitecture free of necrosis and inflammation (Figure 4A). Diabetic Rats' livers showed proliferation of bile ducts, inflammatory changes and necrosis (Figure 4B), while rats treated with crocin (Figure 4C) and Los (Figure 4D), and crocin + Los group (Figure 4E) showed a protective effect against hepatocyte damage by reducing bile duct proliferation and inflammation. The protection observed with crocin + Los appeared to exert remarkable protection against diabetes‐induced liver necrosis.

**TABLE 4:** The effect of crocin and Los on histological alterations of liver tissue.

Groups	Necrosis of hepatocytes	Inflammatory changes	Proliferation of bile ducts	Hepatocytic degeneration
Healthy	—	—	—	—
Diabetic	+2	+2	+2	+1
Crocin	+1	+1	+1	+1
Los	+1	+1	+1	+1
Crocin + Los	—	+1	+1	+1

## 4 Discussion

In addition to the kidney and pancreas, the liver is one of the tissues that is impacted by blood glucose fluctuations [18, 19]. Numerous factors, such as mitochondrial dysfunction and endoplasmic reticulum stress, might affect the development of diabetes complications [20, 21]. RAGE expression and interaction with AGE in liver stellate cells and hepatocytes during a hyperglycaemia situation may stimulate cell growth, oxygen‐free radical production, NF‐Κb activity and upregulate the production of cytokines, growth factors and adhesive molecules that improve fibroblast differentiation [22, 23, 24]. TGF‐β is a potent profibrogenic cytokine and elevated TGF‐β levels as a result of oxidative stress activate profibrotic cells. The increase in TNF‐α expression in hyperglycaemic conditions, on the other hand, plays an important role in the stimulation of hepatic stellate cells, which are involved in the creation of type I collagen in the liver as well as the regeneration of the extracellular matrix and tissue fibrosis of the liver [25]. Therefore, it seems that by reducing the level of glucose and reducing the activity of the AGE‐RAGE axis, and as a result, reducing the production and activity of inflammatory cytokines, the process of liver damage is reduced [26, 27, 28].

In our research, FBG was significantly increased in the diabetes group compared to the healthy group. But, in the group that received crocin and Los at the same time, the level of this biochemical factor decreased significantly. The results obtained from Sepahi et al.'s study showed that crocin can regulate FBG and lipid levels, reduce fat accumulation in the liver and kidney fibrosis and play a role in the AMPK‐dependent autophagy proces [29]. It was also reported in Li et al.'s study that crocin can play a protective role against podocyte damage by inhibiting NF‐κb and reducing glucose [30]. In the findings of Kang et al.'s study, crocin significantly increased insulin sensitivity and decreased serum glucose by acetyl‐CoA carboxylase and MAPK phosphorylation [31]. Also, in this study, serum FBG levels decreased in diabetic rats treated with Los. In fact, by inhibiting the vascular contraction caused by Angiotensin II, Los led to the transfer of glucose to muscle tissue and increased tissue sensitivity to insulin [32]. Studies support the hypoglycaemic effects of crocin and Los [29, 30, 31, 32].

The results of this study showed that serum levels of ALT and AST, which are important markers of liver damage, significantly increased in the diabetic group, indicating liver cell destruction and damage due to hyperglycaemia. This increase may be attributed to oxidative stress and inflammation caused by elevated blood glucose levels, which stimulate harmful pathways in the liver [33]. However, in the groups treated simultaneously with crocin and los, a significant reduction in ALT and AST levels was observed, highlighting the protective role of these compounds in reducing liver damage. Crocin, as a potent antioxidant, mitigates oxidative stress and inflammation [34], while los, by inhibiting the harmful effects of angiotensin II and enhancing insulin sensitivity, led to a significant improvement in liver function [35]. These findings are consistent with previous studies that have confirmed the hepatoprotective effects of crocin and los in diabetic conditions [36, 37].

The results of our study indicated an increased expression of RAGE receptor in the diabetic group, reflecting the activation of pathways associated with oxidative stress and inflammation. The elevated RAGE expression under hyperglycaemic conditions can lead to increased interactions with AGEs, which, through NF‐κB activation, stimulates the production of free radicals and inflammatory cytokines such as TNF‐α and IL‐6 [38]. These pathways contribute not only to cellular damage and liver fibrosis but also exacerbate diabetic complications [39]. However, the significant reduction in RAGE expression observed in the groups treated with crocin, los and their combination demonstrates the protective effects of these compounds through the inhibition of the AGE‐RAGE pathway. Crocin exerts its protective role by inhibiting the NF‐κB pathway, reducing inflammatory cytokine production and improving mitochondrial function [40]. Los helps mitigate the negative effects of AGEs and RAGE by lowering blood pressure and reducing inflammation related to the renin‐angiotensin system [41]. Additionally, crocin combined with los may have synergistic effects, leading to a more effective reduction in RAGE expression. These results are consistent with previous studies, including the research by Pengmin Chen et al. [42], which confirmed the role of the AGE‐RAGE pathway in diabetes and its associated complications.

High levels of AGEs and activation of RAGE can exacerbate fibrotic responses and increase the production of TGF‐β [43]. In other words, inflammation and oxidative stress induced by RAGE enhance TGF‐β signalling, creating a feedback loop that promotes fibrosis [43, 44]. Our results report a significant increase in TGF‐β gene expression in the diabetic group, indicating a connection between RAGE and TGF‐β. TGF‐β plays a crucial role in fibrosis and tissue remodelling, especially in chronic diseases like diabetes [44]. When activated, it leads to extracellular matrix component accumulation and increased collagen production, resulting in fibrosis and organ dysfunction [45]. This study demonstrated that crocin significantly reduces TGF‐β gene expression in liver tissue, which aligns with its ability to combat oxidative stress and lower blood glucose levels [46]. This reduction is likely due to decreased activation of fibrotic pathways, including Smad, mediated by TGF‐β [44, 46]. The present study also found that los significantly reduced TGF‐β gene expression, likely by inhibiting the PI3K/Akt/mTOR signalling pathway and thereby preventing the detrimental effects of TGF‐β [44]. The combination of crocin and los showed a more pronounced effect in reducing TGF‐β expression compared to individual treatments. This synergistic effect likely arises from crocin's antioxidant properties and glucose‐lowering effects combined with los's ability to inhibit angiotensin II and its downstream effects on fibrosis. The combined treatment not only enhances the reduction of TGF‐β expression but also more effectively improves overall liver function. Consequently, crocin and los reduce TGF‐β gene expression through distinct but complementary mechanisms. Such findings underscore the potential of these treatments in mitigating fibrosis and related complications in diabetic conditions.

The results of the present study showed that TNF‐α gene expression was significantly increased in the diabetic group, indicating severe inflammation and overactivity of the immune system [47]. RAGE contributes to the increased production of TNF‐α by activating NF‐κB and MAPK pathways, thereby exacerbating inflammation and oxidative stress [48]. In this study, a significant reduction in TNF‐α expression was observed in the groups treated with crocin los, and the combination of crocin and los, which was consistent with previous studies [49, 50, 51]. This reduction reflects the protective effects of these compounds through inhibition of the AGE‐RAGE pathway and decreased NF‐κB activity. Crocin, by reducing oxidative stress and inflammation [50], and los, by inhibiting the negative effects of angiotensin II and associated inflammation [49, 51], effectively reduced TNF‐α production. The combination of these two agents appears to have synergistic effects and more effectively reduces inflammation and diabetic damage. These results confirm that combined therapies can offer more favourable outcomes in reducing inflammation and improving metabolic status in diabetic patients.

The results of the present study indicate significant alterations in liver tissue structure, particularly in the diabetic group. While the liver in the healthy group exhibited a healthy and homogeneous tissue structure with no necrosis or inflammation, the livers of diabetic rats showed considerable damage, including bile duct proliferation, inflammatory changes and necrosis. These changes suggest the detrimental effects of hyperglycaemia on the liver, leading to tissue damage [52]. In contrast, the groups treated with crocin and los, particularly the combination group of crocin + los, demonstrated a significant reduction in bile duct proliferation and inflammation. These findings indicate that both compounds, especially in combination, provide effective protective effects against diabetes‐induced liver damage and are capable of reducing liver necrosis. The combination of crocin and los appears to have synergistic effects, offering notable protection against diabetes‐related liver damage. These results underscore the importance of using combination therapies in managing liver damage associated with diabetes.

## 5 Conclusions

Crocin could improve liver function and tissue damage by reducing blood glucose and decreasing the expression of TGF‐β, TNF‐α and RAGE. Furthermore, we showed that crocin is able to increase the efficacy of losartan. Thus, crocin may be a promising therapeutic agent for treating diabetes and its consequences when combined with chemical medications. To reach definitive conclusions, human research is still required.

## Author Contributions

A.R.F., Y.M. and S.R. conceived the original idea and designed the study. Y.M., S.R. and H.K.‐R. conducted the experiments. S.R. collected the data. M.R.‐M. performed the liver histopathology. S.R. and A.R.F. analysed the data. S.R. wrote the first draft of the manuscript. All authors reviewed the manuscript.

## Conflicts of Interest

The authors declare no conflicts of interest.

## Data Availability

The data that support the findings of this study are available from the corresponding author upon reasonable request.
